# Study of the Interaction of an Iron Phthalocyanine Complex over Surface Modified Carbon Nanotubes

**DOI:** 10.3390/ma14154067

**Published:** 2021-07-21

**Authors:** María Pérez-Cadenas, Esther Asedegbega-Nieto, Jonathan Carter, James A. Anderson, Inmaculada Rodríguez-Ramos, Antonio Guerrero-Ruiz

**Affiliations:** 1 Departamento Química Inorgánica y Técnica, Facultad de Ciencias UNED, Paseo Senda del Rey No. 9, 28040 Madrid, Spain; easedegbega@ccia.uned.es (E.A.-N.); aguerrero@ccia.uned.es (A.G.-R.); 2 Surface Chemistry and Catalysis Group, Department Chemistry, University of Aberdeen, Aberdeen AB24 3UE, UK; j.carter.05@aberdeen.ac.uk (J.C.); j.anderson@abdn.ac.uk (J.A.A.); 3 Instituto de Catálisis y Petroleoquímica, UA UNED-ICP (CSIC), Calle Marie Curie 2, 28049 Madrid, Spain; irodriguez@icp.csic.es

**Keywords:** carbon nanotubes, iron phthalocyanine, functionalization

## Abstract

Carbon nanotubes (CNT) were prepared by a modified chemical vapor deposition (CVD) method. The synthesized carbon materials were treated with acidic and basic solutions in order to introduce certain surface functional groups, mainly containing oxygen (OCNT) or amine (ACNT) species. These modified CNTs (OCNT and ACNT) as well as the originally prepared CNT were reacted with a non-ionic Fe complex, Iron (II) Phthalocyanine, and three composites were obtained. The amount of metal complex introduced in each case and the interaction between the complex and the CNT materials were studied with the aid of various characterization techniques such as TGA, XRD, and XPS. The results obtained in these experiments all indicated that the interaction between the complex and the CNT was greatly affected by the functionalization of the latter.

## 1. Introduction

Transition metal complexes such as metallophthalocyanines (MPcs) are probably the most flexible organic compounds widely studied in the last decades. These complexes play an important role in various applications and modern technology fields such as in dyes [1], sensors [2,3,4,5], electronic devices [6,7], sensitizers for photodynamic cancer therapy [8,9,10,11], etc. This is in virtue of their excellent optical and electronic properties as well as their chemical and thermal stability due to their π–π stacking [12,13]. The extended delocalized π-electron conjugated macrocyclic system of metallophthalocyanines makes them suitable candidates for their combination with other materials to form composites. These composites potentially combine the unique qualities of both the MPc and the selected support, thereby reducing the inconveniences involved in employing the pure complex. Some uses include the catalysis field where the support-attached phthalocyanine can be employed as a heterogeneous catalyst with the advantage of its regeneration after use, avoiding the limitations caused by the limited solubility of most phthalocyanines and the difficulty in recovering the soluble ones [14,15]. Materials such as zeolites, silica, titania, and alumina are the most widely mentioned support materials in the literature. Other important purposes which have long been considered are in the optimization of film sensing properties. Studies employing Cu phthalocyanine–polymer composites reported that the dispersion of the aggregates of this complex into polymer matrixes enhanced the gas sensing characteristics when compared with the corresponding pure phthalocyanine [16]. The separation of phthalocyanine aggregates by a polymer changes the electrical characteristics and may facilitate admission of gas molecules to the adsorption centers at the appropriate polymer structure. More recently, studies involving carbon nanotubes as support have been being conducted for their application as electrodes, field effect transistors, photoelectric conversion devices, biosensors [4,6,17,18,19,20], etc. These carbon materials are receiving increasing scientific interest due to their exceptional physical properties. Therefore, it is of great importance to conserve these properties when employed as support in composites. The creation of non-covalent π–π interactions between the carbon nanotubes and the macrocyclic compounds would be a viable means of producing composites without causing irreversible damage to their respective electronic structures and, at the same time, would conserve their unique properties. Additionally, and with respect to their catalytic applications, electro catalysis [21] and the possibility to promote the formation of single atom metal supported catalysts [22] can be considered, among others. Moreover, it is a known fact that some transition metal-based pthalocyanines, such as FePc, are active catalysts in various industrial sectors such as in the petrochemical field [23] and in improving electron transfer, which favors the formation of active radical species necessary in various environmental processes [24]. It is known that when considering FePc-CNT composites, the delocalized π electrons would bring about a decrease in the electronic density of Fe. This phenomenon is very interesting in reactions where the presence of metals existing in different oxidation states (as in the case of iron, Fe^2+^/Fe^3+^ redox pair) facilitates reactions where oxidation transitions are required. In a study carried out by González-Gaitán et al., where charge transfer was relevant for ORR reactions, a comparison was carried out employing two different phthalocyanines (FePc and CoPc), and it was observed that the interaction with the CNT was more significant for FePc than for CoPc. This was detected with the aid of characterization techniques whose results inferred that upon interaction with CNT, the Fe atom possessed lower electron density as opposed to what happened in the case of Co, which remained unaltered [25]. In another electrocatalytic study, the activity of composites based on functionalized and non-functionalized CNT combined with FePc was performed, and the highest electron donating effect when the CNT was functionalized with carboxylic acid groups was found [26]. Hence, the presence of these functional groups could enhance or limit the electron donating effect expected between iron phthalocyanine and the CNT. In the present work, carbon nanotubes will be synthesized and functionalized with acid and basic surface groups. The influence of these surface functional groups on the π–π interaction between carbon nanotubes and a covalent iron phthalocyanine will be analyzed.

## 2. Materials and Methods

### 2.1. Synthesis of Carbon Materials

Carbon nanotubes were grown on quartz surfaces using the catalytic vapor decomposition (CVD) method. The procedure consisted of employing acetylene (40 mL/min, Carburos Metalicos, Barcelona, Spain, 99.6%) as the carbon source and Fe(CO)_5_ (Aldrich, St. Louis, MO, USA, >99.99%) as the Fe precursor catalysts, both concealed under an inert nitrogen atmosphere. N_2_ (Carburos Metalicos, 99.9992%) serves two purposes: a smaller flow of 150 mL/min is bubbled through the liquid catalyst precursor and carried into the reactor, while a higher flow of 3850 mL/min is used to transfer all gases into the reactor. A total of 40 mL/min of H_2_ (Carburos Metalicos, 99.9992%) was also introduced in order to provide a reducing atmosphere, thus ensuring that metallic Fe is obtained after the decomposition of Fe(CO)_5_. This synthesis reaction was carried out in a quartz reactor of 125 cm length and 4.5 cm internal diameter, heated in a 66.5 cm furnace (Carbolite furnaces, Sheffield, UK) at a constant temperature of 1023 K. Within the reactor, in the central zone, a 29.9 cm tube containing 16 smaller tubes (14.9 cm long) was placed in order to collect the carbon nanotubes formed. Once cooled overnight under N_2_ flow, the deposited products were retrieved by scratching and subsequently treated with HCl (Panreac, Barcelona, Spain, 37%) and washed with distillated water.

The as-synthesized CNTs were oxidized. For this purpose, they were treated with nitric acid (Panreac, 65%) at 363 K for 72 h. The mixture was maintained under constant stirring, employing a magnetic stirrer. Thereafter, the resulting material was filtered in a Büchner device whose funnel counted with a fritted glass filter (pore size No.4). Vacuum necessary was achieved thanks to a water pump connected to the device. Upon collection, the filtered solid was dried over night at 373 K.

The oxidized nanotubes were further aminated. Amine surface functionalities were introduced to the surface of the oxidized CNTs using a solution of 20% ethylenediamine (EDA) (Panreac, >99%) in n-hexane (Panreac, >99%). This solution was heated to 343 K under constant stirring and held at this temperature for 24 h. Thereafter, the mixture was filtered and dried in a vacuum oven at 373 K for 18 h.

### 2.2. Synthesis of Composities

All three prepared carbon nanotubes were used in the synthesis of composites. In order to obtain a 5% Fe loading, the corresponding amount of Iron(II) pthalocyanine (Alfa Aesar, Haverhill, MA, USA, >95%) was dissolved in 100 mL of n-hexane (Panreac, >99%). The CNTs were added to this solution, and the resulting mixture was stirred for 17 h at room temperature. After this period, the solvent was evaporated, and the material was further dried over night at 373 K to ensure a solvent-free product.

### 2.3. Characterisation

The prepared materials were studied employing various characterization techniques. Transmission electronic microscopy (TEM) was performed on modified and unmodified CNTs employing a JOEL JEM 2000FX system. Surface area and pore size distribution were determined from N_2_ (Carburos Metalicos, 99.9992%) adsorption at 77 K (Micromeritics ASAP 2000 surface analyzer). Thermogravimetric analysis data was collected using a SDTQ600 5200 TA system. In the latter, the samples were heated from room temperature to 1273 K, with a heating rate of 10 K min^−1^ under helium (Carburos Metalicos, 99.9992%). Temperature-programmed desorption (TPD) experiments were performed under vacuum in a quartz reactor coupled with a mass spectrometer (Baltzers, QMG 421), which served as a detection system of the species desorbed from the CNTs during the TPD experiments. The sample was heated to 1100 K at a rate of 10 K min^−1^, with a continuous analysis of evolved gases using the mass spectrometer.

The surface of the CNTs and composites was analyzed by X-ray photoelectron spectroscopy (XPS) with an Omicron spectrometer system equipped with a hemispherical electron analyzer operating in a constant pass energy, using Mg Kα radiation (hν = 1253.6 eV). The samples were fixed to the sample holder using a carbon adhesive tape. The background pressure in the analysis chamber was kept below 5 × 10^−9^ mbar during data acquisition. Survey scan spectra were made at a pass energy of 50 eV, while the C 1s, O 1s, N 1s, and Fe 2p_3/2_ individual high resolution spectra were taken at a pass energy of 20 eV. All binding energies (BE) were referenced to the C 1s line at 284.6 eV.

## 3. Results and Discussion

### 3.1. Carbon Nanotubes

**Figure 1:**
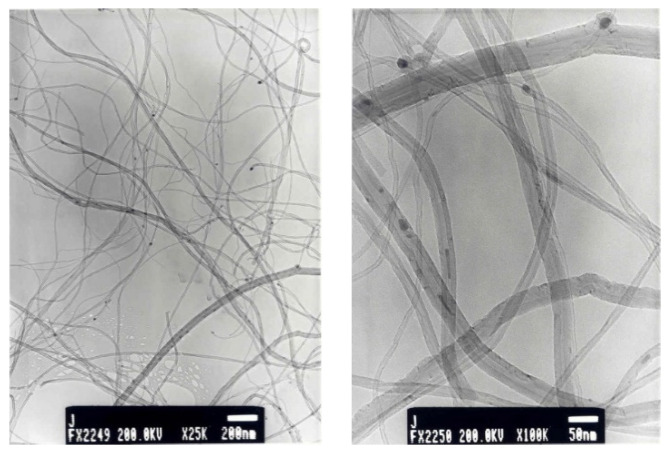
TEM images of untreated CNTs.

Figure 1 shows the TEM images of the synthesized CNTs. Micrographs indicate that all of these materials consist of bundles of tubes of quite different widths. The diameters are generally in the range between 10 and 20 nm.

**Figure 2:**
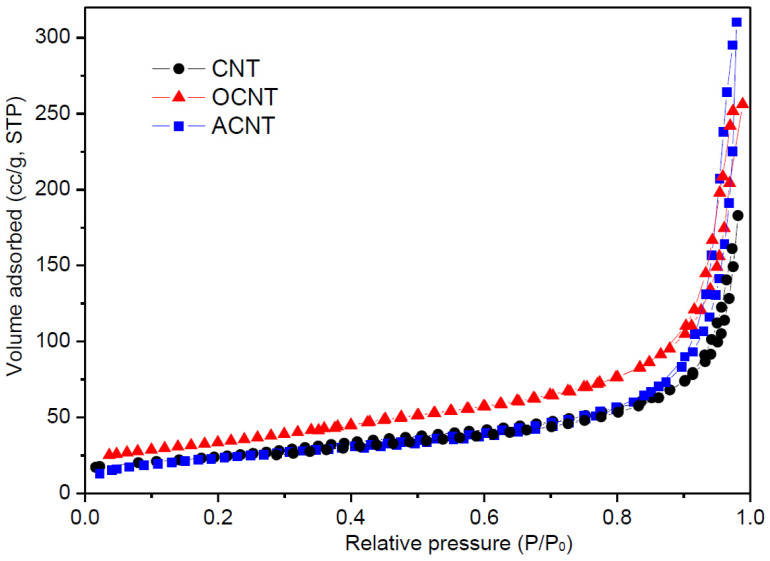
Adsorption-desorption isotherms of N_2_ of the originally prepared CNT, oxidized CNT, and aminated CNT.

Morphological properties were measured by nitrogen adsorption at 77 K. Figure 2 displays the corresponding isotherms. These materials show a type II isotherm, according to the Brunauer–Deming–Deming–Teller, BDDT, classification [27], characteristic of materials with large mesopores and macropores [28]. After oxidation of the originally prepared MWCNTs (Figure 2), the presence of a hysteresis loop at high relative pressures indicates an increase in mesopores (Figure 2). The presence of mesopores in these materials can be either due to cavities within the nanotubes or due to folds between different nanotubes. As can be observed on viewing Figure 2 (adsorption isotherms), there are no significant differences (when comparing pristine and functionalized CNTs) owing to the fact that incorporated functional groups are likely to be at the exterior of the nanotubes, rather than inside the tubes. Hence, the pores remain unaltered.

The originally synthesized CNT sample has a surface area of 90 m^2^/g, which increased to 120 m^2^/g after surface oxidation. This is most likely a result of the elimination of some retained Fe particles at tube ends by the HNO_3_ solution, giving rise to the formation of large mesopores [29,30]. However, after functionalizing of these oxidized CNT surfaces with ethylenediamine, the surface area was lowered to 82 m^2^/g, possibly due to N_2_ adsorption restriction caused by the amine coverage.

The residual weights of all materials after heat treatment under an inert helium atmosphere were determined by TGA. The oxidation of synthesized CNTs gave rise to an increase in weight loss when this material was heated. This increase was higher when this functionalized material was aminated. This confirmed that the modification process was successful.

**Figure 3:**
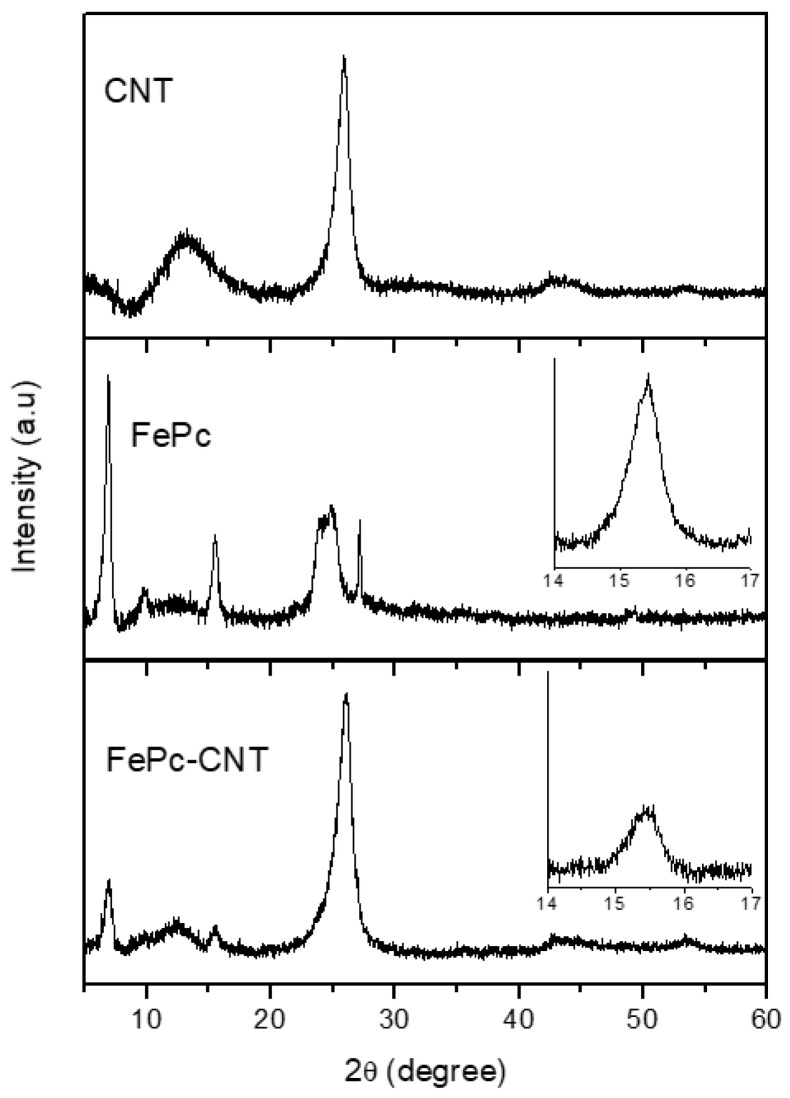
TPD profiles: (**a**) CO_2_ evolution; (**b**) CO evolution. Solid lines for ACNT, dashed lines for OCNT, and dotted lines for CNT.

**Figure 4:**
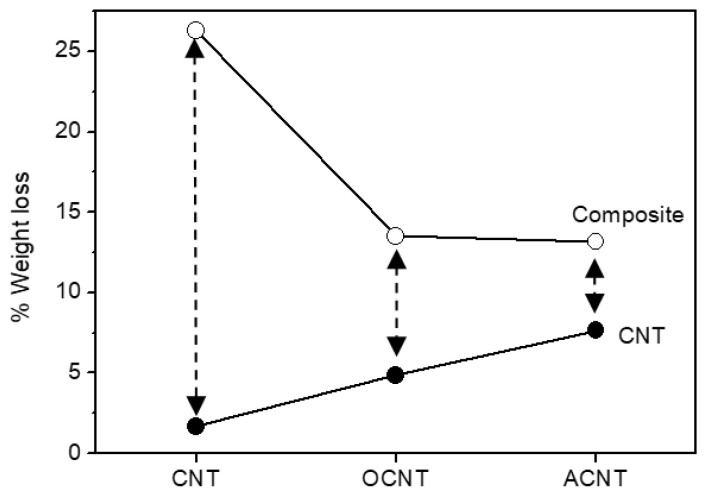
O 1s XPS spectra of (**a**) OCNT and (**b**) ACNT.

XPS gave very valuable information regarding the surface functionalization. Table 1 summarizes the atomic % composition of the different elements present at the surface of the prepared and modified carbon nanotubes. There was a significant increase in oxygen when the CNT sample was oxidized. This proves that oxidation treatment was successful and is in agreement with the increase in desorbed CO and CO_2_ observed from TPD analysis when the CNT sample was oxidized (Figure 3). This also explains the increase in weight loss observed by TGA. The presence of oxygen surface groups in the CNT could be beneficial for the posterior formation of composites as it promotes axial interactions with the complex (in our case FePc), thereby avoiding aggregation and favoring the π–π interactions between the complex and CNT [31]. The atomic % of oxygen was reduced after amination, and, at the same time, the N 1s peak became apparent, confirming the formation of nitrogen groups on the surface, and formed at the expense of the existing oxygen groups. This N 1s peak has a maximum at a binding energy of ca. 400 eV. This value is normally ascribed to amines or amides. According to the literature data, amine species correspond to peak components with BE at 400.2, 399.7 or 400.5 eV, while values of about 399.9 and 399 eV can be identified as amide species [32,33]. The high resolution O 1s spectra of OCNT and ACNT are illustrated in Figure 4. The envelope was deconvoluted into three peaks: binding energy values at about 531.5 eV can be attributed to C=O groups, 532.8 eV is characteristic of C-OH and C-O-C species, and 534.4 eV corresponds to COOH [34]. Carboxylic oxygen groups are greatly reduced from 10% to 6% after amination due to the reaction of these acid groups with the amine of ethylenediamine to form the corresponding amide. 

### 3.2. FePc-CNT Composites

**Figure 5:**
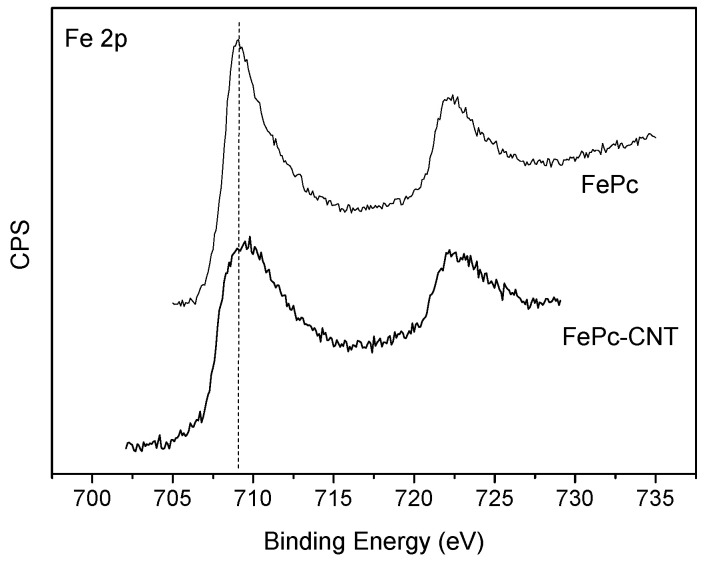
XRD patterns of CNT, FePc and FePc-CNT composite.

The results obtained from the characterization of FePc-CNTs composites gave indications of the type of interaction existing between metallophthalocyanine and carbon support. In the first place, to verify the effectiveness of the incorporation of FePc into the CNTs, XRD before and after the introduction of iron phthalocyanine was carried out. X-Ray diffraction patterns for CNT, OCNT, and ACNT samples are similar to each other, and so, for the sake of brevity, only that of the pristine CNT was presented here. This is shown in Figure 5 together with patterns for the corresponding composite and the pure FePc complex. For CNT, peaks at a 2θ angle of 26° and 43°, associated with the (002) and (100) diffractions of the hexagonal graphite structure [35,36], respectively, were observed. As for the FePc complex, four main peaks at 6.8°, 15.4°, 24.5°, and 27.1° were observed. The FePc-CNT diffraction pattern shows the same characteristic peaks typical of CNT, indicating that its structure was not destroyed after composite formation. Apart from these peaks, other less intense peaks were visible. These appeared at the same 2θ angles as those observed for the pure FePc complex and indicated that the FePc molecule had been incorporated onto the CNT. In order to have a closer view at this, high resolution analysis for the peak at 15.4° was performed for both FePc and FePc-CNT (the patterns are included in Figure 5). This peak was chosen as the most appropriate to determine the presence of the crystalline complex in the composite patterns, as that at 6.8° is at a very low angle and those at higher angles were overlapped by the graphitic peak at 26° corresponding to the CNT. The FePc-OCNT and FePc-ACNT composites showed the same FePc peaks, although their intensity was considerably lower, especially for the ACNT, indicating a poorer incorporation of the complex in this case.

**Figure 6:**
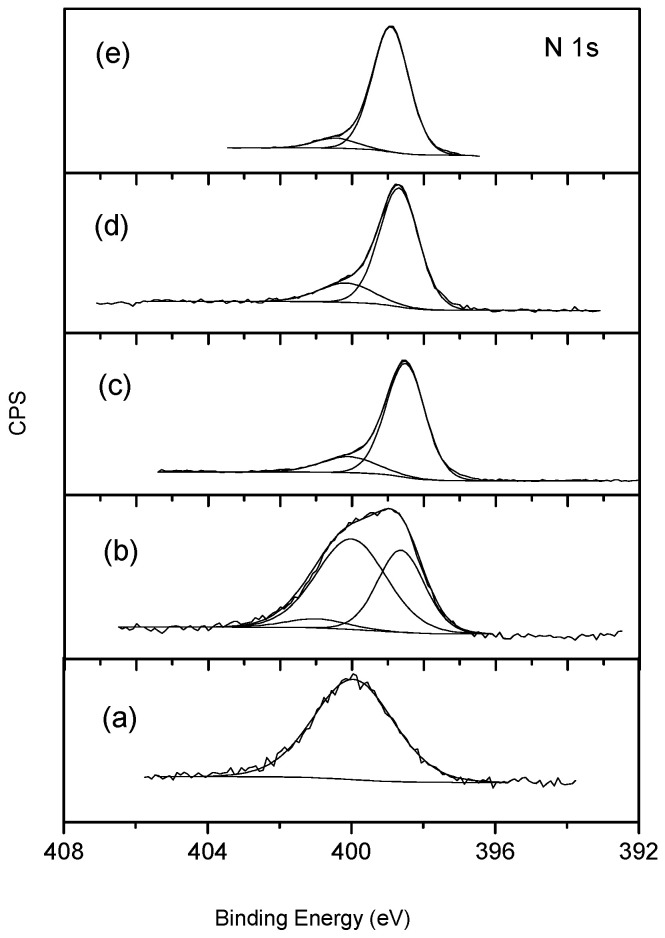
%Weight loss for CNTs and composites at 1000 K.

TGA under nitrogen was carried out on the three complexes. As was expected, there was an increase in weight loss when comparing the composite to the corresponding CNT, owing to the presence of the complex with respect to the CNTs. The difference in %wt loss between CNT and composite should give an estimate of the amount of iron phthalocyanine introduced. This can be better viewed in Figure 6 where the %wt loss of both CNTs and composites at 1000 K is presented. The above defined difference in %wt loss is not constant for all composite CNTs. The highest value (ca. 25%) was observed in the non-functionalized CNT, implying that, in this case, the greatest amount of iron phthalocyanine was present in the composite. There was a vast reduction in the amount of incorporated complex when the originally synthesized CNT was functionalized. Therefore, the introduction of surface groups onto the nanotubes inhibits the interaction with the phthalocyanine, as the value of difference in %wt loss was reduced by a factor of 3.

**Figure 7:**
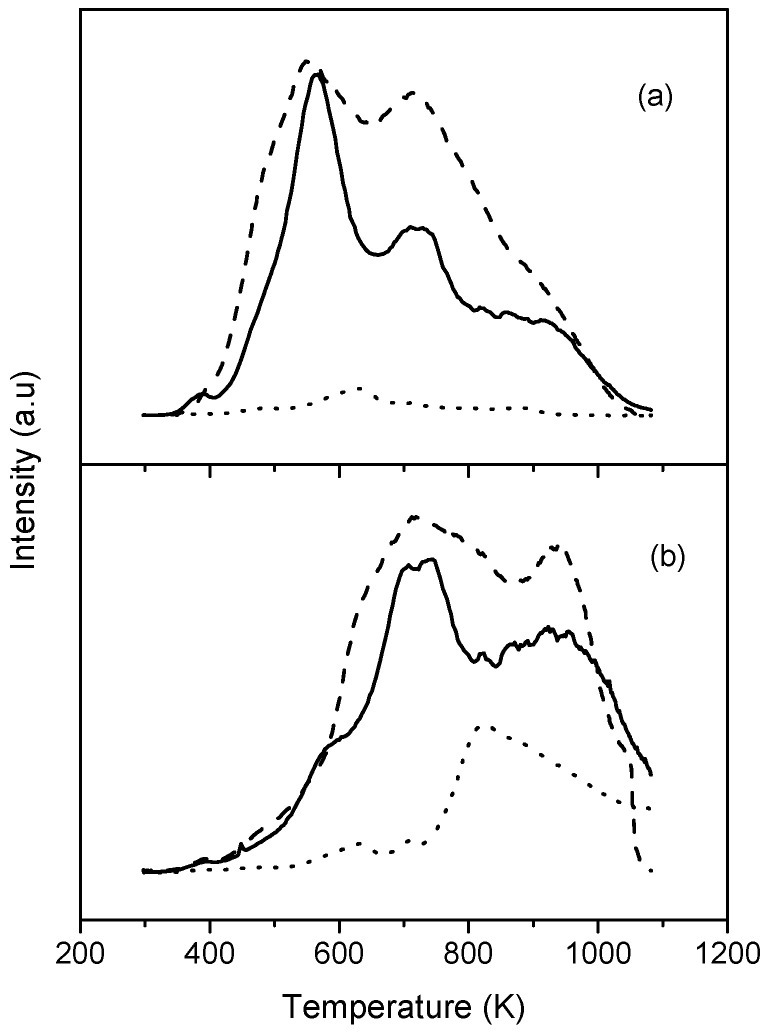
Fe 2p XPS spectra.

The existence of the metallophthalocyanine complex was further confirmed on viewing the XPS results. The iron content at the surface of the obtained composites was evaluated, and the atomic % values found were 0.42, 0.37, and 0.07% for FePc-CNT, FePc-OCNT, and FePc-ACNT, respectively. This suggests that the amount of complex is highest in the non-functionalized CNT, in agreement with the TGA results. At the same time, the iron content at the surface of ACNT was very low, indicating the difficulty in the molecule–substrate interaction when nanotubes are aminated. This is in agreement with the previously discussed XRD and TGA results. XPS of the original FePc was carried out as well in order to determine any change in its oxidation state when adsorbed on the CNT. In Figure 7, the Fe 2p^3/2^ spectra of FePc and one of the composites (FePc-CNT) are depicted. With respect to binding energies, the organometallic complex has a value of 709.4 eV typical of Fe^2+^ species [29]. As for the composites, this value was slightly shifted to higher binding energies (709.8–710 eV), indicating some degree of interaction between the metal ions and the π electrons of the aromatic CNT structures. In this case, the increase in binding energy suggests that Fe acts as an electron donor, while the CNTs are acceptors of the π electrons. In a study involving composites based on this same phthalocyanine and MWCNTs (pristine and modified with -COOH and -NH groups), similar XPS results were obtained as refers to the higher binding energy of Fe (710.9 eV) in comparison with the FePc [26]. This was assigned to Fe (III). This shift to a higher binding energy was also justified as being due to the delocalization of the π electrons, which made iron possess a lower electron density. Similarly, the shift in the binding energy of Fe can also be attributed to columbic interactions between FePc and CNTs [37].

**Figure 8:**
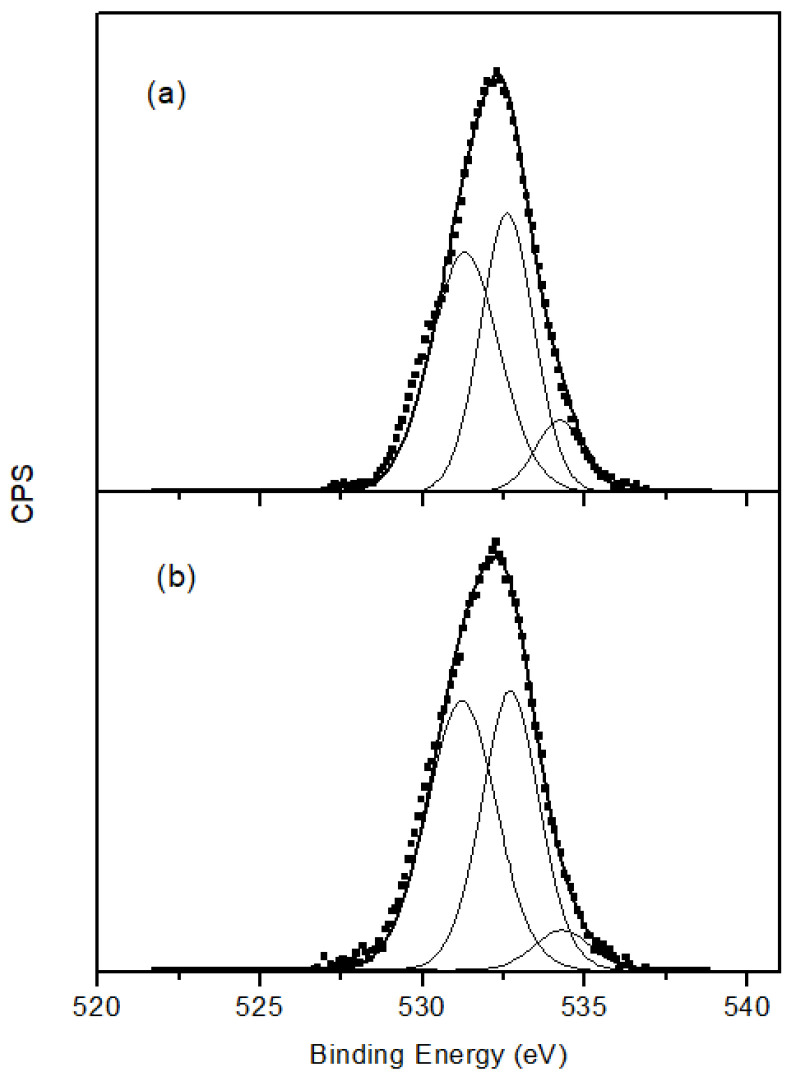
N 1s XPS spectra: (**a**) FePc; (**b**) FePc-CNT; (**c**) FePc-OCNT; (**d**) FePc-ACNT; (**e**) ACNT.

High resolution XPS N 1s was also performed. The deconvolution of this feature is straightforward as has been reported for phthalocyanines [38,39]. It consists of two peaks, one at 399.0 eV due to the two chemically non-equivalent nitrogens (four central nitrogens and four aza nitrogens) and a less intensive peak at 400.6 eV, which can be attributed to a shake-up satellite. Both FePc-CNT and FePc-OCNT show that at these two peaks, the only nitrogen incorporated is that of the phthalocyanine. As can be viewed in Figure 8, their spectra are similar to that of the pure FePc (which has also been presented). As for the FePc-ACNT composite, the peak envelope is quite different in shape and width from that of the other two composites, and apart from the two previously mentioned peaks due to the phthalocyanine, there is a larger peak coincident in both form and binding energy with that observed in the aminated carbon nanotube, ACNT. Therefore, in this composite, nitrogen is present in the phthalocyanine form, although the N species introduced during the amination of the CNT are also present. As indicated in the N 1s atomic ratio compiled in Table 1, only 0.79% belongs to the participation of FePc after subtracting that due to the original ACNT. This value is much lower than that observed for the CNT (6.32%) and OCNT (4.03%) composites. Therefore, N atomic % due to the presence of FePc is highest for the non-functionalized CNT composite, confirming what was already deduced from the Fe 2p XPS result, i.e., that this CNT incorporates the greatest amounts of metallocomplex. This value decreased with the functionalization of CNT and was lowest for the ACNT composite. This is once again in agreement with all other characterization results reported in this study.

Generally, two types of interactions can occur between CNT and the phthalocyanine: π–π interactions between the aromatic components of both materials and interactions due to defects at the edges of the CNT and the phthalocyanine. Various studies carried out in this respect concluded that the principal forces intervening between components are π–π interactions. Some authors point out, after carrying out DFT calculations, that in order to increase the surface of contact and, therefore, enhance π–π interactions, the phthalocyanine undergoes severe distortion [40,41]. In the first of these works [40], the molecular orbitals were described for this non-covalent interaction, and it is said that HOMO is localized on the phthalocyanine, while the LUMO belongs to the CNT. In the second cited reference [41], a comparison is made among carbon structures of different lengths, and it was deduced that this interaction is highest when the dimension of the carbon nanocluster is longer (i.e., CNTs > fullerenes). Jiang et al. even went ahead with more extended planar structures, such as graphene sheets, proving the relevance of π–π interactions between the extended, delocalized electrons belonging to the graphene and the phtalocyanine molecules [42]. As was mentioned in previous paragraphs, the presence of surface groups could enhance this type of interaction thanks to their axial association with the complex, which avoids its agglomeration (since the complex can also undergo π–π stacking between its molecules). At the same time, the shift in the binding energy of iron deduced from XPS also hints at the influence of this π delocalization on the more electropositive Fe species. 

Bearing all this in mind, and considering our experimental results, in our study, we believe that the interaction is mainly between the aromatic ligands of phthalocyanine and the surface of the carbon nanotubes (π–π interactions). We arrived at this conclusion on observing that upon the functionalization of CNT, the amount of FePc is reduced with respect to what occurs with the pristine CNT. Hence, the presence of functional groups does not favor interaction (and therefore incorporation) of FePc.

## 4. Conclusions

The synthesis and consequential functionalization of multiwalled carbon nanotubes were successfully performed using the described procedures. The incorporation of iron (II) phthalocyanine was also achieved, and the amount introduced for the three CNTs, CNT, OCNT, and ACNT, was highly dependent on the functionalization. This was strongly supported by the characterization studies carried out. TGA, XRD, and XPS results showed that FePc incorporation was greatest in the non-functionalized CNT and lowest for the aminated CNT, ACNT. Two types of interactions could exist between both components of the composite: on one hand, π–π interactions between the aromatic species of CNT and FePc. On the other hand, we could also have some interactions due to defects at the edges of the CNT and the phthalocyanine. The results obtained in our study hint at the existence of the first-mentioned interaction. At the same time, it was deduced that the presence of the functional groups inhibits contact between the CNTs and the complex, hindering their π–π interactions, resulting in a reduction in the extent to which FePc may be introduced. Apart from these π–π interactions, the shift in the Fe 2p_3/2_ binding energy suggested the existence of some interaction between metal ions and the π electrons of the aromatic structures of CNTs (π-d complexes). As regards the nature of the CNTs, adsorption isotherms indicate that the incorporation of functional groups is achieved at the exterior surface (and not inside the tubes), as there are no significant differences between the mesoporous properties of all CNTs studied (as-synthesized and modified). 

## Figures and Tables

**Table 1:** Atomic % composition as determined by XPS.

Sample	C 1s	O 1s	N 1s
CNT	97.50	2.36	0.00
OCNT	92.16	7.69	0.00
ACNT	93.74	4.08	2.12
FePc-CNT	90.28	2.98	6.32
FePc-OCNT	89.94	5.66	4.03
FePc-ACNT	92.40	4.62	2.91

## Data Availability

Data are contained within the article.
